# The Opportunity Cost of Family Caregiving: New Directions for Policy and Practice Solutions

**DOI:** 10.1093/geroni/igaf122.591

**Published:** 2025-12-31

**Authors:** Selena Caldera

**Affiliations:** AARP, Washington, District of Columbia, United States

## Abstract

This presentation details a new study from AARP providing updated national estimates of the opportunity cost of family caregiving. The Urban Institute’s Dynamic Simulation of Income Model was calibrated using the latest Caregiving in the US data on the demographics, care tasks, and economic characteristics of family caregivers. This model examines counterfactual scenarios for adults who stopped or limited labor force participation to provide care to another adult with disabilities or a health condition requiring regular assistance. The study estimates potential lost income, lost savings, and lost Social Security income for caregivers who stop working altogether and for those who reduce their work hours to accommodate their care responsibilities. Disparate outcomes for caregivers of different age groups, income levels, and levels of care intensity will be summarized. This detailed analysis is critical to understanding that there is no one-size-fits-all solution to financially support family caregivers. Instead, a range of solutions targeted to the experiences and needs of different groups of caregivers is needed. These findings are crucial for developing targeted policy and practice solutions that mitigate family caregivers’ potential financial losses. In conclusion, a range of innovative policy and practice solutions to protect the financial security of family caregivers will be discussed.

